# Platelet Rich Plasma Therapy in Achilles and Patellar Tendinopathies: Outcomes in Subjects with Diabetes (A Retrospective Case-Control Study)

**DOI:** 10.3390/jcm13185443

**Published:** 2024-09-13

**Authors:** Michele Abate, Roberto Paganelli, Raffaello Pellegrino, Angelo Di Iorio, Vincenzo Salini

**Affiliations:** 1 IRCSS Ospedale San Raffaele, 20132 Milan, Italy; mic.abate@icloud.com (M.A.); salini.vincenzo@hsr.it (V.S.); 2 Saint Camillus International University of Health and Medical Sciences, 00131 Rome, Italy; roberto.paganelli@unicamillus.org; 3 Department of Medicine and Surgery, LUM University, 70010 Casamassima, Italy; r.pellegrino@lum.it; 4 Department of Innovative Technologies in Medicine and Dentistry, University “G. d’Annunzio” Chieti-Pescara, Viale Abruzzo 322, 66100 Chieti, Italy

**Keywords:** platelet rich plasma, diabetes, tendinopathy, mini-invasive

## Abstract

**Introduction:** Diabetes mellitus (DM) is associated with a high risk of chronic degenerative Achilles (AT) and Patellar (PT) tendinopathies and ruptures. Growth factors (GFs) synthesis in diabetics is substantially decreased in human connective tissues, including in tendons. Platelet Rich Plasma (PRP), which is enriched in GFs, might prove of great help in tendon healing. The aim of the study was to assess whether pre-existent DM or Impaired Glucose Tolerance (IGT) could influence the clinical outcome in subjects undergoing PRP treatment. **Methods:** Sixty subjects with diabetes/pre-diabetes and sixty euglycemic controls, matched for sex and age, were enrolled. Patients suffering from proximal insertional PT and mid-portion AT, treated with PRP therapy, were included in the study. To assess the basal status and the efficacy of the therapy after 3 and 6 months, the Victorian Institute of Sport Assessment (VISA) questionnaire and the Ultrasound methodology study were used. Patient satisfaction was assessed by means of the Likert Scale. **Results:** In the population study at 6 months, the mean VISA-score increased (8.92 ± 0.67; *p*-value < 0.001). The improvement in the diabetic group was less evident compared to the controls (−2.76 ± 0.95; *p*-value = 0.003). Even though the improvement was poor, it was still significant. MCID analysis revealed that diabetics had higher risk of therapeutic unsuccess. Logistic regression analysis was applied to assess factors associated with unsatisfactory results (Likert-scale) of PRP treatment: AT (O.R.: 3.05; 95%CI: 1.40–6.64; *p*-value = 0.005), higher BMI values (O.R.: 1.02; 95%CI: 1.01–1.04; *p*-value = 0.01), and lower VISA score values at baseline (O.R.: 0.95; 95%CI: 0.90–0.99; *p*-value = 0.04). **Conclusions:** PRP treatment in AT and PT chronic tendinopathies resulted in less favorable results in subjects with diabetes compared with euglycemic subjects. Moreover, the subjects with PT showed better results than those with AT.

## 1. Introduction

Type II diabetes mellitus (DM) is associated with high risk of tendinopathy. The tendons of these subjects show significant histopathological alterations (disorganized collagen fibers, microtears, fibro-chondral metaplasia, and calcifications), increased stiffness and thickness, and impaired biomechanical properties [1,2]. From the clinical point of view, individuals with DM are four times more likely to experience chronic tendinopathy or a tendon tear or rupture than euglycemic controls [3,4,5]. This is a challenging health care problem for at least two reasons: (1) the increasing prevalence of subjects with diabetes and pre-diabetes in the western population; (2) the frequent habit of diabetics to practice sports activities, which are useful for controlling the metabolic disorder, but at the same time submit tendons to overuse and therefore expose them to an additional risk of tendinopathy.

Complex mechanisms are involved in the pathogenesis of tendon damage. These mechanisms can be grouped into three main categories: increased storage of Glycation End Products (AGEs), high glucose environment, and dysregulation of neuropeptide signaling. AGEs accumulate in tissues and form cross-links with targeted proteins, which are nearly irreversible once formed; specifically, in tendons, AGEs lead to an increase in collagen cross-links, and therefore to a reduced fiber sliding and increased stiffness [6]. AGEs also play a role in the erroneous differentiation of Tendon Derived Stem Cells (TDSCs) to the chondrocyte and osteoblast lineages, thereby contributing to the mechanisms of ectopic chondro-ossification [7]. The binding of AGEs to their specific receptors (RAGE) triggers pro-inflammatory cytokines release (IL-6 and TNF-α), increases apoptosis, enhances oxidative stress, inhibits nitric oxide synthase (NOS) activity, and quenches released NO (v6). The high glucose environment inhibits proliferation, causes apoptosis, and suppresses the expression of tendon related markers in TDSCs [8]. Finally, the dysregulation of neuropeptide signaling can negatively affect tendon homoeostasis, mainly influencing the expression of cytokines and growth factors (GFs), inflammation, and immune responses [9]. Indeed, GFs synthesis (mainly VEGF, insulin growth factors (IGFs), and neurotrophins) is substantially decreased in human connective tissues, such as dermis and tendons [9,10]. 

Besides the increased risk of tendon damage, the patients with DM exhibit delayed and/or defective healing responses following tendon surgical repair, characterized by the formation of scar-tissue rather than the regeneration of native tendon structure [11,12,13]. Unsatisfactory post-operative blood glucose control is significantly associated with an increased risk of rotator cuff retears [14,15]. As far as conservative treatments for tendinopathies are concerned, several therapeutic approaches have been suggested, using drugs (hyaluronic acid, steroids, polidocanol, etc.) [16] or physical therapies (eccentric exercise, laser therapy, shock wave therapy, percutaneous electrolysis, etc.) [17] with controversial results. In particular, the clinical experience suggests that these therapies have a reduced efficacy in subjects with diabetes. However, this has not been tested in specifically designed studies, in particular on the outcomes of Platelet Rich Plasma (PRP) therapy [18]. 

Therefore, we hypothesized that since GFs synthesis is substantially decreased in tendons of diabetic patients [9,10], PRP, which is enriched in GFs [19], might prove of great help in tendon healing, as it does in the treatment of diabetic foot ulcers [20,21]. 

Considering that to our knowledge, no information is available on this issue, we performed an analysis investigating whether pre-existent DM or Impaired Glucose Tolerance (IGT) could influence the clinical outcome in subjects undergoing PRP therapy for Achilles and Patellar tendinopathies. A secondary objective of the study was to identify factors associated with better clinical results.

## 2. Materials and Methods

### 2.1. Type of Study

This is an observational retrospective case-control study, performed in subjects with Achilles and Patellar tendinopathies treated with PRP. The research was performed in accordance with the ethical standards as laid down in the 1964 Declaration of Helsinki and its later amendments or comparable ethical standards; the approval of the IRB of the University “G. D’Annunzio” Chieti was acquired (CRRM:2024;01;10;01). Informed consent for retrospective epidemiological study inclusion was routinely obtained from all individuals prior to treatment.

### 2.2. Patients 

Data of patients suffering from proximal insertional Patellar and mid-portion Achilles tendinopathies for more than 3 months, treated with PRP therapy between 2015 and 2018, were selected from the Orthopedic Outpatients service database of the University G. D’Annunzio and included in the study. All cases were evaluated following a standardized protocol. The diagnosis of tendinopathy was made in relation to history, clinical examination (pain, tenderness, and functional limitation), and ultrasound (US) features of tendon damage. Exclusion criteria were the following: (a) Achilles or Patellar tendons tears or history of tendon surgery; (b) recent knee and ankle trauma; (c) treatments within the previous three months with steroids, hyaluronic acid, and/or physical therapies (e.g., exercise, laser, extra-corporeal shock wave, ultrasound). Other exclusion criteria were joint dysmorphisms and biomechanical changes, rheumatic pathologies (rheumatoid, psoriatic, and reactive arthritis, arthritis associated with inflammatory bowel diseases, and spondylarthritis), severe systemic diseases (renal, hepatic, cardiac, infections, endocrinopathies, malignancies), immunodepression, anticoagulant or antiaggregant therapy, Hb values < 11 g/dL, and/or platelet values < 150,000/mm^3^. The diagnosis of diabetes and pre-diabetes was made according to the guidelines of the American Diabetic Association [22]. For each diabetic or pre-diabetic patient, a subject with the same tendinopathy, without the metabolic disorder (normal fasting glucose and Hb1aC values), matched for sex, age (±3 years), duration of symptoms (±4 months), and VISA score (±5 points), was selected as control.

### 2.3. Measures

At baseline, the following data were collected: (a) sex and age; (b) symptoms of tendinopathy and their duration (in months); (c) medical history (diseases, blood examinations, drugs assumption); (d) sport practice; (e) smoking and alcohol consumption. Current smokers were considered to be those subjects who smoked more than three cigarettes/day. Alcohol consumption was considered when more than two units/day were consumed. Tendon pain and function were evaluated by means of the Victorian Institute of Sport Assessment- Patellar (VISA-P) and Achilles tendon (VISA-A) questionnaire (adapted to the Italian language) [23]. The VISA questionnaire assesses functional evaluation of Patellar and Achilles tendon and consists of eight tasks that measure pain and function in daily living and sports activity. Results range from 0 to 100, where 100 represents the perfect score.

Height and weight were measured, and BMI was then calculated. Blood pressure was measured at rest three times at 5-min intervals, and the mean was computed. Hypertension was diagnosed for systolic blood pressure > 140 mm Hg and diastolic blood pressure > 90 mm Hg. Finally, venous blood was collected to measure blood cells, hemoglobin, glycated hemoglobin (Hb1aC), triglycerides, total cholesterol, and HDL cholesterol. 

### 2.4. US Evaluation

Participants underwent an ultrasound (US) and Color Doppler (CD) evaluation of the affected tendon, using a high-resolution, multi-frequency (6–15 MHz) linear array transducer (ProSound ALPHA10, Aloka, Japan). In line with standard protocols, both longitudinal and transverse scans were taken [24]. Patellar insertional tendinopathy was diagnosed when focal tendon thickening, abnormal tendon echotexture, enthesophytes (step up bony prominence at the end of the normal bone contour), and bone erosions (cortical breakage with a step-down contour defect) were observed. Similarly, the presence of inhomogeneous hypo- or hyperechoic thickening of the tendon, diffuse or focal, along with a loss of the normal pattern of fibers and irregular edges of the tendon margins, was considered as sign of midportion Achilles tendinopathy. According to a priori classification, suitable for clinical purposes, based on the structural abnormalities, tendons were then stratified for severity as “mild” (one area of disorganized echotexture i.e., focal inhomogeneous area with loss of fibrillar pattern), “moderate” (more than one areas of disorganized echotexture i.e., inhomogeneous hypo- or hyperechoic tendon damage with altered fibrillar pattern), and “severe” (diffuse disorganized echotexture and hypo- or hyperechoic areas with irregularity of tendon margins and/or calcifications) [25]. The presence of neovascularization was assessed by means of Color Doppler and graded as (0), (1+), (2+), or (3+) according to a semi-quantitative estimate of the number of vessels. When no vessels were visible, the estimate was 0. When one or two small vessels were visible, mostly in the anterior part of the tendon, the estimate was (+1). When several irregular vessels were observed throughout the tendon, the estimate was (+2) to (+3) [26]. To avoid artifacts, sensitivity was optimized for low flow, and the gain was set just below the noise level.

### 2.5. Therapeutic Procedure

After the clinical assessment, patients were submitted to Platelet Rich Plasma treatment, which was performed in sterile conditions and under US-guidance. PRP was obtained from 8 mLS of autologous blood using the RegenKit^®^ A-PRP device provided by RegenLab (Le Mont-sur-Lausanne, Switzerland), which, according to the manufacturer (single spin, 3500 rpm for 5 min) provides an average 3× native platelet concentration, >80% platelet recovery, no leukocytes, red blood cell remnant <0.3%. After local anesthesia with 10 mL of mepivacaine 2%, a 21G needle was inserted into the degenerate tendon and small autologous PRP deposits were left at the site of the most damaged areas, and then proximal and distal (peppering technique), for a total amount of 4–5 mL. Three injections of PRP were performed weekly. After each injection, patients were kept under observation for approximately 30 min (monitoring early side effects) and then discharged home. At home, patients were instructed to abstain from intense activities for 3–4 days; moreover, they were asked to register possible adverse events (pain, swelling, heat, functional limitations). If necessary, ice packs and acetaminophen (but not NSAIDs) were allowed. 

### 2.6. Follow-Up

After PRP therapy, a rehabilitation program based on eccentric training and stretching specific for Achilles and Patellar tendons was implemented. Exercises were gradually allowed within the first three weeks after PRP injections and performed for at least three months, during which a gradual increase of physical activity was encouraged. The home-based exercises were as described by Alfredson et al. [26] (3 × 15 repetitions twice daily); they were performed at slow speed, and load was increased when pain was absent. The clinical and US assessments were repeated at 3 and 6 months after the last injection. The US evaluations and the therapeutic procedures were performed by the same well-trained operator (AM). Finally, the post-treatment patient satisfaction was assessed by means of the Likert Scale (0 = not at all satisfied; 1 = slightly satisfied; 2 = somewhat satisfied; 3 = very satisfied; 4 = extremely satisfied) [27].

### 2.7. Statistical Analysis

The baseline anthropometric and clinical characteristics of the subjects belonging to the two experimental groups (subjects with diabetes and non-diabetic controls) were compared using analysis of variance and chi-square test according to linear and to categorical variables, respectively. To test whether diabetes was associated with the rate of improvement after PRP treatment (i.e., the increase in VISA total score) at 3 and 6 months, we performed linear mixed models (LMM) analysis; VISA total score was considered as the independent variable (fixed effects) in different models; moreover, age, sex, and BMI were considered in the models as potential confounders. Second-order analyses were also performed to verify the multiplicative effect of the interaction for the time of the study, and time; moreover, as random effects, intercept and slope, with unstructured covariance, were also considered in the models. Model A reported the unconditional means model, which evaluated just the random effect for the intercept without any predictors; Model B reported the unconditional growth model, which considered the effect of time; and Models C also included a second-order analysis with an interaction term; all models were also adjusted for age, sex, and BMI. Akaike’s information criterion (AIC) was used to examine improvements in model fit; for all studied models, smaller values represent better fitting models. The following estimates were derived from the analysis: γ00 = intercept of the average trajectory; γ01 = intercept of the trajectory for diabetes diagnosis; γ10 = slope of the average trajectory; γ11 = slope of the average trajectory for time*diabetes diagnosis; ρ^2^_e_ = within-person variance components; 20 = in initial status variance components; ρ^2^_1_ = in rate of change in variance components [28]. Lastly, to assess factors associated with unsatisfactory results of PRP-injection treatment (Likert < 1), a logistic regression model was applied. The independent factors were the site tendon treated, BMI, VISA score, and diabetes diagnosis. To assess the unsuccess risk for diabetic patients, we used logistic regression analysis, where the dependent variable was the change of the VISA-score between baseline to last follow-up, and the Minimally Clinically Important Differences (MCID) cut-off was settled at a score greater or equal to 6.5; in a second model, the MCID cut-off was settled to a score greater or equal to 20 points. Lastly, to assess the risk of therapeutic unsuccess considering three levels variables, where both MCID level were categorized simultaneously, we used generalized estimating equations (GEE) analysis with a multinomial option. SAS version 9.4 for Windows (SAS Institute, Inc., Cary, NC, USA) was used for all data processing and statistical analyses. We set the level of statistical significance at *p* < 0.05 (2-tailed).

## 3. Results

In total, 60 subjects with diabetes/pre-diabetes and 60 euglycemic controls, matched for sex and age, were enrolled. In each study group, 32 (53.3%) people with AT and 28 (46.7%) people with PT were included. No statistical difference between groups for demographic parameters, hypertension, smoking, alcohol consumption, or sport activities was observed (Table 1). 

According to selection criteria, the clinical features (mean VISA score) were similar in both groups. A longer duration of tendon symptomatology, even if not clearly significant (*p* = 0.06), was observed in subjects with diabetes. Mean BMI was higher (*p* < 0.001) in the hyperglycemic group, as well as the mean values of Hb1aC, total cholesterol, and triglycerides (Table 1). On the contrary, HDL values were higher in euglycemic controls. Sonographic texture abnormalities were detected in all participants. Severe texture changes were more frequent in diabetics (44.3% vs. 28.3%) but the difference did not reach the level of statistical significance. On the contrary, a trend to a higher percentage of neo-vessels grade 3 was found in controls (6.67% vs. 20.0%). After PRP treatment, only a few subjects reported adverse events, mainly pain, swelling, and/or heat at the point of injection, which quickly regressed after ice pack application and acetaminophen administration. Unfortunately, this information was missing in several reports, and so the percentage of adverse events could not be exactly quantified. 

**Figure 1:**
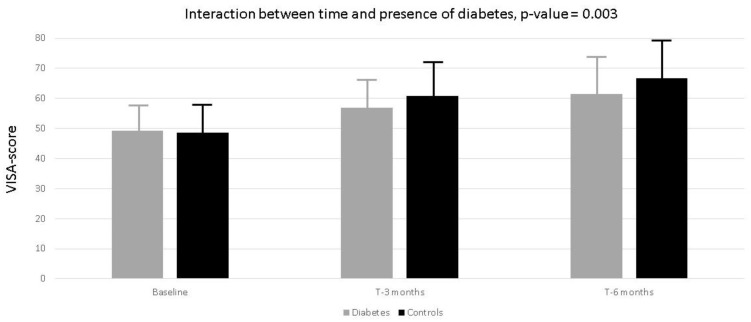
Linear Mixed Model analysis. Mean variation of the VISA-score through times of the study according to presence of diabetes (grey) and non-diabetic patients (black). The second order *p*-value for the interaction between times of the study and diabetes was reported. The LMM complete model was reported in the Supplementary Table S1.

In Figure 1, the changes of VISA score in the different follow-up periods are reported. In both groups, an increase of the score was observed, but it was less evident in diabetics (Supplementary Materials Table S1). Indeed, the VISA increase across time was 8.92 ± 0.67, independent of the disease status (Supplementary Materials Table S1, γ 10: *p*-value < 0.001). In the diabetic group, as demonstrated by the interaction between time for disease status, the increase across time was less evident compared to controls (Supplementary Materials Table S1, γ11; β ± SE: −2.76 ± 0.95; *p*-value = 0.003). Interestingly, the increase of the VISA score at 6 months was more evident, even if not statistically significant (β ± SE: 2.15 ± 1.37; *p*-value = 0.12) in the subjects with Patellar Tendinopathy, compared to the subjects with Achilles Tendinopathy, in both groups. 

**Figure 2:**
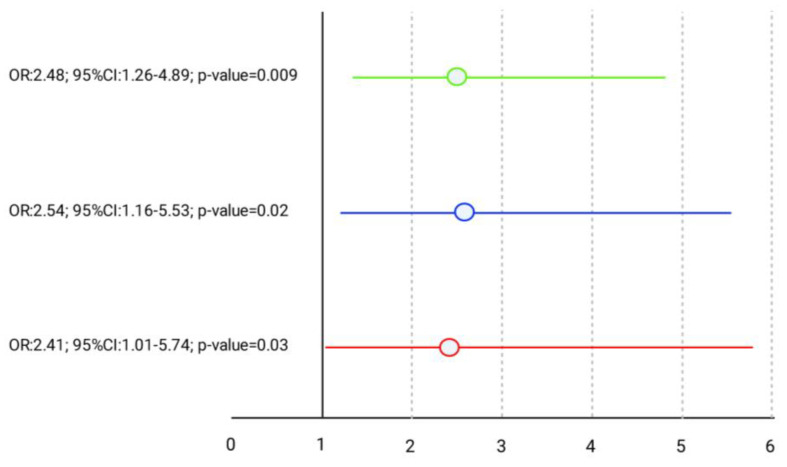
Logistic regression analysis: Odds ratio (OR), and 95% confidence interval (95%CI) for the treatment unsuccess according to disease status in different cut-off points for VISA changes over time. In red, we reported the OR and 95%CI in the MCID with a cut-off greater or equal to 6.5 points increase in the VISA; in blue, we reported MCID with a cut-off greater or equal to 20 points increase. In green, we show the multinomial analysis that incorporates both cut-offs.

Moreover, treatment failure risk according to MCID for VISA-score is also evident in Figure 2. When MCID was settled to a value greater or equal to 6.5 points at the end of the study, 27% of the enrolled subjects did not reach this threshold, and diabetic subjects showed a higher risk, OR = 2.41 (95%CI: 1.01–5.74, *p*-value = 0.03). 

When the threshold for the VISA-score change was set at greater or equal to 20 points, 81 subjects (66.4%) did not reach the MCID, and again diabetic subjects were more prone to a higher risk of poor outcome (OR2.54; 95%CI: 1.16–5.53; *p*-value = 0.02). We assessed the risk of therapeutic failure for diabetics in three levels (multinomial) according to the VISA-score MCID cut-off (6 to 19, and greater or equal to 20). In this case, diabetic subjects had a higher risk of worse results compared to euglycemic subjects. In order to assess which factors could be predictive for such increased risk in diabetics, we analyzed the MCID as a multinomial logistic function; among the metabolic markers, only Hb1aC was significantly associated with poorer outcome, independent from age (OR: 1.16 95%CI:1.06–1.29; *p*-value = 0.003), sex (OR: 0.42; 95%CI: 0.13–1.31; *p*-value = 0.14), BMI (OR: 1.01 95%CI:0.99–1.04; *p*-value = 0.22), and site of tendinopathy (where AT was the reference, OR: 6.38; 95%CI:1.95–20.89; *p*-value = 0.003), and for each unit increase in Hb1aC the risk of failure showed an OR: 1.09 (95%CI: 1.04–1.14, *p*-value < 0.001). Finally, we evaluated which parameters could be related to the patients’ satisfaction (Likert scale) after PRP treatment, dividing the subjects in two groups (a-unsatisfied—score 0–1; b-somewhat/very/extremely satisfied—score 3–5). During univariate analysis, the subjects who were more satisfied showed the following basal characteristics: (1) presence of patellar tendinopathy (*p* < 0.001); (2) lower BMI values (*p* < 0.02); (3) higher total VISA score at 6 months (*p* < 0.04). Age, symptoms duration, hypertension, smoking and drinking habits, and practice of sport activities were not associated with satisfaction (Table 2), neither the score of sonographic pathologic features or the presence of neo-vessels. Logistic regression analysis confirmed the association of unsatisfactory results of PRP treatment with Achilles Tendinopathy (O.R.:3.05; 95%CI: 1.40–6.64; *p*-value = 0.005), higher BMI values (O.R.:1.02; 95%CI:1.01–1.04; *p*-value= 0.01), and lower VISA score values (O.R.:0.95; 95%CI: 0.90–0.99; *p*-value = 0.04). Interestingly, no association was observed between diabetes and the patients’ satisfaction (Table 3). 

## 4. Discussion

The main finding of this real-world study of a cohort of patients is that PRP treatment for Achilles and Patellar chronic tendinopathies achieves less satisfactory results in subjects with diabetes in comparison to euglycemic ones, as previously suspected by anecdotal reports. 

To discuss this finding, it is worth remembering that the efficacy of PRP in the treatment of tendinopathies gives conflicting results in different studies. While some authors claim satisfactory outcomes [29], others do not report significant improvements in trials where PRP was matched against placebo [30]. Several factors may be invoked to explain this inconsistency, such as the tendon treated, the type and stage of the tendinopathy, the number and schedule of injections, the PRP procedure and therefore composition, the internal milieu, and the concomitant physical activity [30]. 

In patients with diabetes, PRP treatment has a sound rational basis, since in these subjects GFs synthesis in tendons is substantially decreased [29]. However, it is evident that, in our subjects, the persistent negative action of the metabolic impairment (increased AGEs and fasting glycemic values), and probably the more severe degenerative damage, hindered the positive effects of GFs contained in PRP. However, it cannot be excluded that the types and amounts of GFs in diabetics are far less than those present in euglycemic persons, thus reducing the effect of PRP. 

Another important point from our results is that DM reduced the efficacy of PRP treatment, not only considering the VISA score but also when taking into account the MCID. Lacking an agreement for a cut-off point to define MCID, with proposed thresholds varying from a minimum of 6.5 points to a maximum of 20 [31,32,33], the response rate to treatment differs, and in our study it ranged from 73% to 36% according to the two suggested cut-offs. Independent of the cut-off chosen (6.5 or 20 points), the risk of treatment failure in diabetic patients was very similar (OR > 6.5 = 2.41, and OR > 20 = 2.54), and more than doubled in both logistic models of analysis. Analyzing the MCID as a multinomial function, the risk for diabetic patients remained unchanged, despite a change in the precision of the estimates, influencing only marginally the confidence intervals.

In diabetic patients, multinomial logistic regression identified glycated hemoglobin as a risk factor among markers of impaired metabolism, independent from age, sex, body composition, and site of tendinopathy. In a recent report, diabetes with poor glycemic control (Hb1aC > 8.5%) had an impact on different components of the platelet transcriptome, which may reflect a subclinical platelet activation in uncontrolled diabetic patients [34]. The authors suggested a mechanism of downregulation of some miRNAs (DLK1–DIO3 gene region on chromosome 14q32.2) that may be a general feature of DM indirectly influencing platelet reactivity [34]. Overall, there is evidence that platelets response and function may be modified by DM, and even more by poor glycemic control, associated with a more pronounced reduction in platelets function and sensitivity to drugs, e.g., aspirin [35]. 

All these results suggest a more cautious approach to tendinopathy in diabetic patients, and glycemic control should be considered to increase the probability of therapeutic success in those patients. 

A new finding in our study is that the subjects with Patellar Tendinopathy are more likely to experience satisfactory results than those with Achilles Tendinopathy, as indicated by the Likert scale data. A probable explanation may be that walking and, even more, running put an increased load on AT (as much as eight times the body weight), submitting the tendon to a greater stress and possibly limiting the perceived therapeutic effect of GFs. This hypothesis is indirectly supported by the finding that a higher BMI is independently associated with reduced satisfaction after therapy. In overweight persons, besides the negative effects of the overload, bioactive peptides (adipokines, cytokines, prostanoids, and metalloproteinases) may act as prolonged disruptors of tendon healing [31]. Tendon is a mechanosensitive tissue, and repeated overloading (in sport and work settings) can lead to collagen fibrils damage, weakening the tendon [36]. Moreover, in DM, tendinopathy is the result of chronic metabolic degeneration, rather than related to injury of a normal tendon [37].

Our study, with a retrospective design, included the assessment of joint dysmorphisms and biomechanical changes during the clinical visit by an expert physiatrist, and those conditions could be ruled out in enrolled patients. 

Finally, findings from the sonographic study deserve a specific mention. It is well known that this methodology can provide morphological information that is very useful for the clinical characterization of tendinopathies [38]. In this framework, we observed a trend towards more frequent severe sonographic texture changes in diabetics, whereas a higher percentage of grade 3 neo-vessels was seen in controls. These findings, which are in keeping with previous observations on diabetic tendinopathy, may be ascribed to a reduced expression of VEGF in connective tissues of diabetic subjects [38].

## 5. Limitations

Several limitations of the present research must be acknowledged. The retrospective nature of the study could have introduced a selection bias, even if we have considered all patients present in the archive according to our selection criteria. The relatively short duration of the follow-up precludes the knowledge of what happened after the sixth month of observation, but our follow-up length is similar to other studies and longer follow-ups in an outpatient setting are difficult to acquire. The lack of a blinding study design could have influenced the outcome, but several concerns could arise, for example in the use of a randomized design, where blindness could be guaranteed, in the use of a placebo-injection. A structured collection form in the feedback of adverse events is lacking, therefore a recall bias must be accounted for, even if a clinical visit was made at every follow-up and presumed adverse events were researched. As for DM, no information was available on the glycemic control during the study period, and this factor might have influenced the final result. Finally, no measurements of the different GFs in the blood of the subjects were performed. Indeed, it must be considered that, in a clinical setting, these important shortcomings are difficult to overcome. 

## 6. Conclusions

This study shows that the efficacy of PRP therapy in diabetic subjects with lower limbs tendinopathies is less evident than in euglycemic controls. Moreover, poor metabolic control (glycated hemoglobin) is associated with the doubled increased risk of therapeutic failure in diabetic patients compared to controls, and the subjects with patellar tendinopathy show better results than those with Achilles Tendinopathy. Taking into account the limitations of the study, PRP treatment in patients with DM should be reserved only for selected cases. 

## Figures and Tables

**Table 1:** Demographic and clinical data at baseline according to disease status. Data were reported as mean ± sd, and absolute number and percentage.

	Diabetes	Controls	
Number	60	60	*p*-value
Male sex	39 (65.00)	39 (65.00)	0.81
Tendons			0.99
Achilles	32 (53.33)	32 (51.61)	
Patellar	28 (46.67)	28 (46.67)	
Age	57.45 ± 6.27	57.52 ± 6.31	0.95
Months symptoms	15.23 ± 4.73	13.61 ± 4.64	0.06
Months diabetes onset	22.43 ± 26.47		
Hb1AC	7.50 ± 1.63	4.51 ± 0.75	<0.001
Total cholesterol	175.47 ± 29.30	154.20 ± 21.14	<0.001
HDL cholesterol	50.50 ± 10.92	61.67 ± 13.60	<0.001
Triglycerides	167.58 ± 49.85	132.22 ± 35.19	<0.001
BMI	26.08 ± 3.10	24.45 ± 1.81	<0.001
Hypertension	15 (25.00)	13 (21.67)	0.66
Smoke	14 (23.33)	8 (13.33)	0.16
Alcohol	11 (18.33)	14 (23.33)	0.50
Running	16 (26.67)	21 (35.00)	0.32
Sport	18 (30.00)	20 (33.33)	0.69
VISA at the enrollment	49.27 ± 7.33	48.65 ± 8.51	0.67
Score US			0.22
1	16 (26.67)	19 (31.67)	
2	18 (30.00)	24 (40.00)	
3	26 (43.33)	17 (28.33)	
Neo-vessels			0.14
0	40 (66.67)	33 (55.00)	
1	6 (10.00)	8 (13.33)	
2	10 (16.67)	7 (11.67)	
3	4 (6.67)	12 (20.00)	
Likert scale			0.08
0	14 (23.33)	16 (26.67)	
1	16 (26.67)	18 (30.00)	
2	19 (31.67)	13 (21.67)	
3	10 (16.67)	5 (8.33)	
4	1 (1.67)	8 (13.33)	

**Table 2:** Demographic and clinical data at baseline, according to unsatisfactory/satisfactory result (Likert <1 or >2) after six months of PRP treatment. Data were reported as mean ± sd, and absolute number and percentage.

	Likert ≤ 1	Likert ≥ 2	
Number	64	56	*p*-value
Male sex	38 (59.38)	40 (71.43)	0.17
Tendons			0.001
Achilles	43 (67.19)	21 (37.50)	
Patellar	21 (32.81)	35 (62.50)	
Age	57.94 ± 6.51	57.00 ± 6.10	0.42
Months symptoms	14.88 ± 4.28	13.84 ± 5.23	0.24
BMI	25.82 ± 2.58	24.68 ± 2.62	0.02
Hypertension	18 (28.13)	10 (17.86)	0.19
Smoke	11 (17.19)	11 (19.64)	0.73
Alcohol	14 (21.88)	11 (19.64)	0.77
Running	21 (32.81)	16 (28.57)	0.62
Sport	22 (34.38)	16 (28.57)	0.50
VISA at the enrollment	47.66 ± 8.17	50.51 ± 7.45	0.04
VISA 3 months	54.60 ± 9.14	63.25 ± 7.95	
VISA 6 months	55.97 ± 7.46	73.34 ± 8.31	
Score US			0.25
1	15 (23.44)	20 (35.71)	
2	26 (40.63)	16 (28.57)	
3	23 (35.94)	20 (35.71)	
Neo-vessels			0.80
0	37 (57.81)	36 (64.29)	
1	7 (10.94)	7 (12.50)	
2	10 (15.63)	7 (12.50)	
3	10 (15.63)	6 (10.71)	
2	10 (16.67)	7 (11.67)	
3	4 (6.67)	12 (20.00)	

**Table 3:** Logistic regression; factors associated with unsatisfactory result (Likert < 1) after six months of PRP-injection treatment.

	O.R.	95%CI	
Tendons			
Achilles	3.05	1.40–6.64	0.005
Patellar	Reference		
BMI	1.02	1.01–1.04	0.01
VISA score	0.95	0.90–0.99	0.04
Diabetes	1.88	0.82–4.33	0.10

## Data Availability

Data will be available on reasonable request.

## Supplementary Materials

The following supporting information can be downloaded at: https://www.mdpi.com/article/10.3390/jcm13185443/s1, Table S1: Mixed Model: Analysis of variation of VISA during the follow-up times. When in the model were considered age, sex, and BMI, and site of treatment, the fit (AIC) of the model decreased, therefore were not reported.
