# Supplementary material for: Platelet Rich Plasma Therapy in Achilles and Patellar Tendinopathies: Outcomes in Subjects with Diabetes (A Retrospective Case-Control Study)
Source: J Clin Med. 2024 Sep 13;13(18):5443. doi: 10.3390/jcm13185443 (PMC11432448; doi:10.3390/jcm13185443)
Supplement: Supplementary file 1 [file jcm-13-05443-s001.zip › jcm-3156711-supplementary.pdf]

Supplementary Table S1: Mixed Model: Analysis of variation of VISA during the follow-up times. When in the model were considered age, sex, and BMI, and site of treatment, the fit (AIC) of the model decreased, therefore were not reported.

			Model A Unconditional Means Model	Model B Unconditional Growth Model	Model C Interaction
Initial status	Intercept	γ_{00}	57.24±0.76 ***	42.16±0.99 ***	40.76±1.38 ***
	No Diabetic	γ_{01}			2.81±1.96
	Diabetes				Reference
Rate of change	Intercept (time)	γ_{10}		7.54±0.49 ***	8.92±0.67 ***
	Interaction time*diabetes	γ_{11}			-2.76±0.95 **
Level 1	Within person	δ^2_e	99.46±9.08 ***	27.07±3.49 ***	27.07±3.49 ***
Level 2	In initial status	δ^2_0	36.04±9.43 ***	53.48±17.12 ***	51.51±16.90 **
	In rate of change	δ^2_1		15.58±4.15 ***	13.68±3.92 ***
	Covariance	δ_{01}		-13.91±7.39 *	-11.68±7.14
		AIC	2771	2538	2530

* p-value<0.05; ** p-value<0.01; *** p-value<0.001

γ_{00} = intercept of the average trajectory; γ_{01} = intercept of the trajectory for diabetes diagnosis; γ_{10} = slope of the average trajectory; γ_{11} = slope of the average trajectory for time*diabetes diagnosis; δ^2_e = within-person variance components; and δ^2_0= in initial status variance components; δ^2_1 = in rate of change in variance components.
